# The Future of IVF: The New Normal in Human Reproduction

**DOI:** 10.1007/s43032-021-00829-3

**Published:** 2022-01-03

**Authors:** Vitaly A. Kushnir, Gary D. Smith, Eli Y. Adashi

**Affiliations:** 1 Department of Obstetrics and Gynecology, University of California Irvine, 333 City Blvd, W. Ste. 1400, Orange, CA 92868 USA; 2 Departments of Molecular and Integrative Physiology, Obstetrics and Gynecology, Urology, University of Michigan, 4422 MS1 1301 E, Catherine St. Ann Arbor, MI 48109 USA; 3 Medical Science, Medicine and Biological Sciences, Brown University, 222 Richmond Street, Providence, RI 02903 USA

**Keywords:** In vitro fertilization, Microfluidics, In vitro gametogenesis, Reproductive genetics

## Abstract

Increased demand for in vitro fertilization (IVF) due to socio-demographic trends, and supply facilitated by new technologies, converged to transform the way a substantial proportion of humans reproduce. The purpose of this article is to describe the societal and demographic trends driving increased worldwide demand for IVF, as well as to provide an overview of emerging technologies that promise to greatly expand IVF utilization and lower its cost.

## Introduction

Since its clinical introduction in 1978, in vitro fertilization (IVF) has redefined the ability of the human species to procreate. Initially developed to aid the infertile couple, clinical indications for IVF have since rapidly expanded to include medical and genetic conditions, as well as fertility preservation. While IVF access and utilization vary widely globally, the practice now accounts for the conception of over 5% of all newborns in some European countries where IVF is more affordable and/or is covered by insurance [1]. The corresponding figure presently stands at 4.1% in Australia and New Zealand, 1.9% in the USA, and 1.7% in China and is rapidly rising in all regions of the world [2, 3]. Infertility, which affects approximately 10% of couples, remains the main driver of IVF utilization. These simple statistics suggest that IVF utilization may significantly grow in the coming decades if barriers to its utilization are lowered; this is without even considering an increasing number of indications for IVF beyond infertility.

Changing demographics and societal norms are driving increased IVF utilization. Improved access of women to educational and career opportunities, as well as effective contraception has contributed to progressively delayed childbearing and overall lower fertility rates worldwide. In many countries and in virtually all US states, fertility rates are now substantially below population replacement levels of 2100 births per 1000 women. In a growing number of metropolitan areas as well as in entire highly developed countries, the average age at first birth now exceeds 30 years, that is, well beyond peak fertility which occurs in the mid 20s. Inadvertently, a growing proportion of women is delaying childbearing to a point where age-related fertility decline contributes to the prevalence of infertility and to increased demand for fertility treatments including IVF and oocyte cryopreservation. These trends will likely accelerate due to the socio-economic impact of the COVID-19 pandemic, which has forestalled new family formation. Indeed, preliminary data from Chinese cities indicate that birth rates declined between 9 and 32.6% in the second half of 2020 compared with 2019, reflecting effects of the COVID-19 lockdowns [4]. Declining fertility rates in China have prompted its government to reverse a decades old one-child policy in favor of a two-child policy in 2016, and to a three-child policy in 2021.

The utilization of IVF is closely linked to its affordability and accessibility [5]. Indeed, a growing number of countries and US states are adopting various policies intended to reverse declining fertility rates. These policies range from legally mandated insurance coverage for fertility treatments to subsidies intended to ease the burdens of child-rearing. The concept that *fertility is a basic human right* is just starting to gain traction and is sure to accelerate wider adoption of such policies [6]. Another recent development is the growing number of prominent corporations opting to fund fertility benefits as a part of their social mission and as a means of attracting and retaining employees. Combined, the various policies that promote improved insurance coverage are bound to lower the cost of IVF to patients and increase its utilization.

The distribution of established fertility clinics thus closely corresponds to affluent metropolitan areas with the lowest fertility rates and the most advanced maternal ages at birth. Conversely, less densely populated and less affluent areas are characterized by relatively poor IVF access. Moreover, racial and ethnic disparities in the utilization of IVF, largely due to socio-economic factors, are inversely correlated with fertility rates [7]. An additional driver of IVF utilization is the growing societal acceptance of non-traditional families including single and same-sex parents. Finally, third-party IVF that includes the use of donor oocytes, sperm, or embryo and gestational carrier is rapidly growing, now accounting for over 20% of all birth conceived through IVF in the USA [8].

The IVF process is complex and stressful, it consists of multiple steps which can take up to several months to complete. The main reasons patient prematurely drop-out of IVF prior to achieving a pregnancy are the financial, physical, and psychological burdens of the treatment regimen [9]. Here, we describe promising future approaches and technological innovations which might improve IVF accessibility while reducing its costs and burden of care.

## Medical Advancements

Controlled ovarian hyperstimulation (COH) is performed to increase the number of oocytes available for IVF. COH involves multiple injections of gonadotropins and serial visits to the fertility clinic for the conduct of transvaginal ultrasound evaluations and the measurement of circulating hormone levels. It follows that COH is complex, time sensitive, and intensive. Various strategies intent on reducing the number of injections by utilizing long-acting gonadotropins or oral medications are already available and are gaining increased acceptance in the field for the treatment of select patient populations [10, 11]. Similarly, an emerging strategy to measure salivary estradiol levels may help decrease the need for blood draws during COH [12]. Recent advancements in portable lower cost ultrasound devices may further simplify follicular and endometrial monitoring by way of convenient mobile facilities and potentially even self-operated endovaginal telemonitoring [13]. Combined, these approaches may greatly simplify COH by rendering it less invasive and by decreasing the time commitment required. Finally, interventions which may further decrease the treatment burden may include screening of patients for psychological issues as well as offering counseling and coping interventions such as e-therapy as an integral part of IVF [14, 15].

## Technological Advancements

Perhaps the most promising technological development that might democratize IVF access in the near-term is the automation and miniaturization of the IVF laboratory. Building, staffing, and manually operating an IVF laboratory account for much of the high cost, maldistribution in access, and variability of outcomes. The basic steps in the IVF laboratory include:

1. identification and separation of sperm and oocytes
2. fertilization
3. embryo culture
4. embryo selection for transfer
5. cryopreservation of surplus embryos and gametes

Great progress has already been made towards the automation of these individual steps by way of new technologies. Still, the IVF process in its entirety remains highly manual. The altogether novel IVF lab-on-a-chip concept has the potential to revolutionize IVF by enabling the automation of virtually all of the steps involved in a single system [16–18].

Microfluidics is defined as a multidisciplinary field of study and design whereby fluid behaviors are accurately controlled and manipulated with small scale geometric constraints that yields dominance of surface forces over volumetric counterparts. Past procedures in the IVF laboratory, though successful, apply a macroscale approaches to microscale cellular biological events [18]. Integration of microfluidics into the IVF laboratory may give rise to at least four foreseeable advantages: (1) precisely controlled fluidic gamete/embryo manipulations; (2) providing biomimetic environments for culture; (3) facilitating microscale genetic and molecular bioassays; and (4) enabling miniaturization and automation. The basic utility and advantages of individual microfluidic devices for gamete and preimplantation embryo isolation, manipulation, and assessment have been demonstrated [18]. Current efforts are focused on integrating extant individualized microfluidic procedural components into a future IVF lab-on-a-chip.

Microfluidic sperm-sorting devices [19–21] and automated sperm analyzers [22] are already being introduced into routine IVF practice. Indeed, microfluidics has been used for the isolation of sperm from semen and testicular biopsies [23–29]. These novel sperm-isolating microfluidics devices provide for the collection of highly motile sperm populations replete with enriched normal morphology, and most importantly, reduced DNA fragmentation relative to conventional methods of sperm isolation [19, 27, 30, 31].

Microfluidic in vitro insemination has been demonstrated [32], whereas conventional fertilization is suitable for the vast majority of IVF patients, microfluidic systems may further decrease the need for Intracytoplasmic Sperm Injection (ICSI). Such outcomes may even be possible in the setting of oligospermia, because even a low concentration of sperm may still be sufficient to achieve fertilization [32]. As ICSI has become a dominant method of insemination in human clinical IVF, the importance of precise microfluidic push/pull cumulus-oocyte-complex cumulus cell removal has been shown to yield good visualization of the oocyte cytoplasm/orientation [33]. The fertilization step by ICSI is perhaps the most technically difficult step to achieve on a commercial scale, but feasibility of one such system has been demonstrated [34]. Future automated ICSI will likely involve a combination of microfluidics, robotics, and refined optics [34, 35].

Embryo culture has already been fully automated with use of time-lapse incubators which allow continuous monitoring of embryo development. Data generated from time-lapse incubators can be analyzed with machine learning to aid in the selection of embryos with the highest pregnancy potential [36–38]. Additional information about embryo viability may be gleaned from other omics technologies which can either sample the embryo directly or indirectly via its culture media. The technologies in question include genomic, transcriptomic, proteomic, and metabolomic analyses [39]. Although the use of preimplantation genetic testing (PGT) of trophectoderm cells of blastocyst stage embryos is quite common in clinical practice, the utility of such testing for the ascertainment of aneuploidy remains controversial on both biological and technical grounds [40]. Microfluidics technology has been successfully used to culture mammalian preimplantation embryos from the zygote to the blastocyst stage both individually and in groups [41–46]. These experiments have proven informative to overcoming the hurdles of microenvironment manipulations in microfluidic devices involving microchannels [42], microfunnels [45], microwells [44], and microdroplets [46] that can induce shear stresses and osmotic shifts that can be detrimental to embryo development [45, 47]. The importance of individual embryo culture in microfluidic devices can be appreciated when one considers the desire to integrate real-time imaging and morphometrics [48], molecular [49], and/or metabolomic [50, 51] bioassays, biomechanics [52], and non-invasive PGT of cell-free DNA in spent media [53]. Noninvasive PGT, which utilizes cell-free DNA released into the spent embryo culture media, is likely to become the first omics technology used clinically in conjunction with a microfluidic system [53].

Finally, cryopreservation of sperm, oocytes, and embryos has become the standard of care. Vitrification has become the dominant method for oocyte and embryo cryopreservation. While semi-automated/automated systems for oocyte/embryo vitrification have been reported and are now in early stages of clinical adoption [54–56], these devices do not necessarily use or require microfluidics. If one looks to the future of a microfluidic automated lab-on-a-chip, the question arises of whether or not microfluidics is useful and/or beneficial in the vitrification process? Microfluidics can be used to precisely control cryoprotectant exposures (gradual versus step-wise exposure) to oocytes/zygotes/embryos and thus reduce osmotic strain, reduce sub-lethal membrane damage, and improve subsequent development [57–60]. Future potential benefits of integrating microfluidics with vitrification and automation have been carefully enumerated in recent reviews [59, 61, 62]. Integrated microfluidics for vitrification with automation is promising. Such a system/device will reduce reagent consumption, decrease labor intensity, facilitate ease of use, offer medium to high throughput, and may foster point-of-care cryopreservation and/or promote in-office cryopreservation procedures that require less in the way of technical/personnel expertise and sophisticated laboratory/equipment needs.

**Fig. 1:**
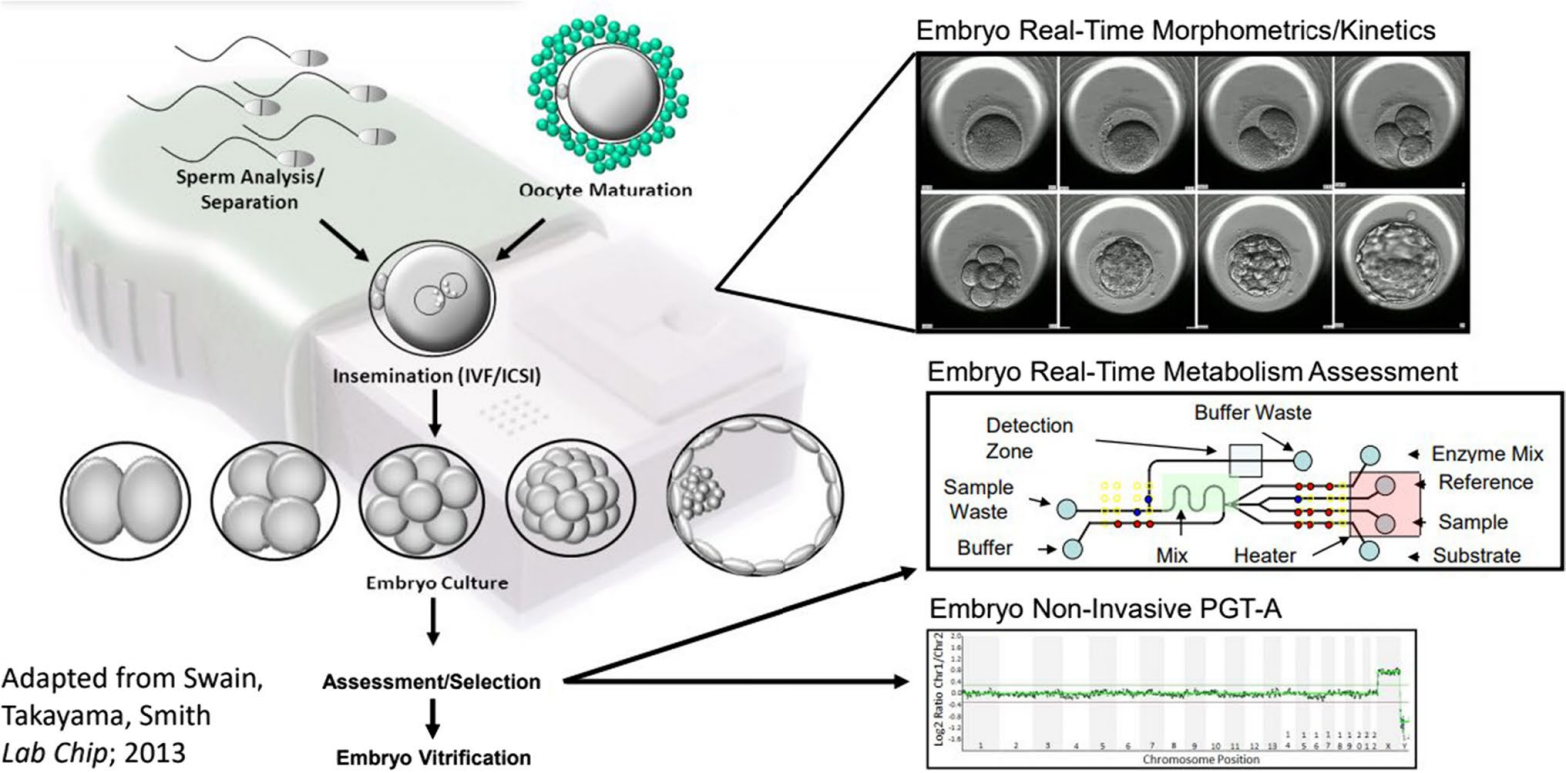
Future IVF lab-on-a-chip concept displaying the integration of all the steps performed during the IVF process and of emerging non-invasive techniques of embryo assessment

Figure 1 illustrates the future IVF lab-on-a-chip concept, including all of the laboratory steps performed during IVF while integrating emerging non-invasive techniques of embryo assessment. Adoption of automated IVF systems offers multiple potential advantages: standardization of workflows, reduction in errors, reduction in cost, reduction in contamination, and the potential for incremental system improvement via machine learning. Additionally, miniaturization and automation of the IVF laboratory can greatly improve accessibility to IVF treatment for underserved communities, especially those who are economically disadvantaged and who reside in rural areas. Regulatory approval will doubtlessly be required if automated systems are to be adequately validated to produce clinical outcomes superior to those attained with the current manual process in the IVF laboratory. Furthermore, automation will likely significantly decrease the staffing requirements and alter the type of skills required to operate fertility centers. It is likely that the technical aspects of IVF will be gradually assumed by machines. This may well increase the emphasis placed on human interactions which supports the medical and psychological needs of patients during their fertility journey.

## Scientific Advancements

Fertility preservation research has steadily increased our understanding of the mechanisms that govern folliculogenesis [63]. The development of in vitro culture systems for follicles provided insights into the relationship between oocytes and their surrounding somatic cells, as well as the requisite hormones and growth factors. Multi-step culture systems have advanced to a point where primordial follicles residing in ovarian cortical tissue can undergo activation, growth, and in vitro maturation (IVM) to yield metaphase II (MII) oocytes [64]. These advancements are expanding fertility preservation via ovarian tissue cryopreservation and subsequent chance at parenthood via IVF to pre-pubertal girls and young women at-risk to develop primary ovarian insufficiency (POI) due to gonadotoxic chemotherapy for cancer or due to other serious diseases. Intriguing extensions of this technology may enable the isolation of oocytes from patients who have already developed POI or have entered natural menopause so long as some dormant follicles remain within their ovarian cortex. Another avenue of research is to develop an artificial ovary as has been achieved in a murine model using 3D printed scaffolds for tissue engineering [65, 66]. Microfluidic culture systems may also be utilized to support follicle development while mimicking the natural menstrual cycle [67].

## In Vitro Gametogenesis (IVG)

Perhaps the most revolutionary concept in modern reproductive science is that of in vitro gametogenesis (IVG). IVG comprises various approaches, including organ culture systems, embryonic stem cells (ESC), induced pluripotent stem cells (iPSC), and spermatogonial stem cells (SSCs). Several of these approaches led to the creation of functional gametes in rodent models [68]. Japanese scientists, who have been at the forefront of IVG research, have recently succeeded in extending these techniques to the generation of human oogonia from iPSCs [69]. Yet, another approach to IVG involves reconstruction of functional oocytes by nuclear transfer of the first polar body genome from an MII oocyte into an enucleated donor MII cytoplasm [70]. This latter technique may well increase the number of oocytes available for the treatment of infertility of women with few or poor-quality autologous oocytes.

The existence of human oogonial stem cells (OSCs) capable of giving rise to new oocytes has been an area of some controversy for nearly a decade. Reports to the effect that cells isolated from human ovarian tissue using fluorescence-activated cell sorting and an antibody against the DDX4 protein constituted OSCs challenged the long-standing dogma that the ovarian reserve is finite [71, 72]. Multiple follow up studies by several groups were unable to confirm the presence of OSCs in the human ovary. Recently, single-cell analysis of the human ovarian cortex failed to identify OSCs [73]. Instead, cells captured by the DDX4-directed antibody proved to be perivascular cellular elements [73].

SSCs constitute the progenitor cells in the process of spermatogenesis. As such, these cells are the focus of in vitro spermatogenesis (IVS) and in vivo restoration of male fertility. While IVS has been achieved in rodent models, it has proven far more difficult to realize in primate counterparts [74]. One recent approach to IVS involved the culture of SSCs with immortalized Sertoli cells. Meiosis and the production of spermatid-like cells followed, albeit in the face of improper activation of cognate meiotic checkpoints [75]. In yet another approach, sperm nuclear transfer allowed production of androgenetic haploid embryonic stem cells which were able to “fertilize” oocytes and support early embryonic development, diploid blastocysts, and ESC generation [76]. Once fully realized, IVS is destined to offer genetic parenthood via IVF to infertile men diagnosed with azoospermia and pre-pubertal boys undergoing gonadotoxic treatments.

## Reproductive Genetics

The convergence of IVF with reproductive genetics has been at the forefront of the field for the past few decades. The development of next generation sequencing has expedited the adoption of PGT of embryos with an eye toward detecting the presence of chromosomal abnormalities. Moreover, increased use of carrier screening of infertile couples has increased the use of PGT for monogenic diseases. As cost of carrier screening decreases and the number of detected mutations expands, a substantial new population of patients identified as carriers may pursue IVF with PGT to build their families. Indeed, population genomic screening of young adults may offer significant healthcare savings through the prevention of rare disorders and cancers [77]. Future applications of PGT may expand to multifactorial diseases and whole-exome screening, though current attempts at introduction of embryo selection based on polygenic scores into clinical practice seem premature and fraught with ethical challenges [78]. Recent improvements in micromanipulation techniques and the development of CRISPR-Cas9 gene editing tools [79] raise the prospect of germline genome modification (GGM) for severe monogenic disorders. Indeed, GGM has already been achieved in human embryos [80]. Mitochondrial replacement therapy (MRT) for the prevention of heritable mitochondrial DNA diseases is even further developed than GGM, with clinical trials already underway in the UK [81].

## Conclusion

The growing utilization of IVF will transform the way a substantial proportion of the human species procreates. It is likely that in the near future, as many as 10% of all children will be conceived through IVF in many parts of the world. Given the rapid scientific and technological evolution of IVG and of reproductive genetics, it is imperative that both the public and regulatory bodies be engaged in establishing a framework for the ethical evaluation of emerging technologies [82–84]. Such public engagement is critical. The absence of such may well result in reactionary bans against clinical research as has been the case for GGM and MRT in the USA [85]. Moreover, the introduction of innovative technologies into clinical practice must be rooted in science and supported by well-designed clinical trials [86]. Premature commercialization of costly and unproven “add-ons” to IVF has been an ongoing issue in the field, ranging from procedures to medicines to laboratory techniques [87, 88]. Collectively, routine application and marketing of unproven IVF add-ons may erode the public trust in the reproductive medicine field. Thus, it is imperative for the field to prioritize requiring confirmation of safety and efficacy of technologies before allowing them to be offered routinely to IVF patients. Reproductive medicine, and especially IVF, is rapidly transforming human reproduction and is thus bound to remain of fundamental importance to both science and society.

## Data Availability

Not applicable.

## Abbreviations

COH: Controlled ovarian hyperstimulation
ESC: Embryonic stem cells
GSC: Germline stem cells
GGM: Germline genome modification
iPSC: Induced pluripotent stem cells
ICSI: Intracytoplasmic sperm injection
IVF: In vitro fertilization
IVG: In vitro gametogenesis
IVM: In vitro maturation
IVS: In vitro spermatogenesis
MII: Metaphase II
MRT: Mitochondrial replacement therapy
OSC: Oogonial stem cells
PGT: Preimplantation genetic testing
POI: Primary ovarian insufficiency
SSC: Spermatogonial stem cells
